# Association of markers of endothelial dysregulation Ang1 and Ang2 with acute kidney injury in critically ill patients

**DOI:** 10.1186/s13054-016-1385-3

**Published:** 2016-07-03

**Authors:** Cassianne Robinson-Cohen, Ronit Katz, Brenda L. Price, Susanna Harju-Baker, Carmen Mikacenic, Jonathan Himmelfarb, W. Conrad Liles, Mark M. Wurfel

**Affiliations:** Kidney Research Institute, Division of Nephrology, Department of Medicine, University of Washington, 325 9th Ave, Box 359606, Seattle, WA 98104 USA; Department of Biostatistics, University of Washington, Seattle, WA USA; Division of Pulmonary and Critical Care Medicine, Department of Medicine, University of Washington, Seattle, WA USA; Center for Lung Biology, Division of Allergy and Infectious Diseases, Department of Medicine, University of Washington, Seattle, WA USA

**Keywords:** Acute kidney injury, Endothelial dysfunction, Endothelial dysregulation, Sepsis

## Abstract

**Background:**

The role of endothelial dysregulation with acute kidney injury (AKI) in critically ill patients is unclear.

**Methods:**

We retrospectively assessed the associations of AKI with biomarkers of endothelial function and inflammation among 948 subjects admitted to the intensive care unit (ICU) at Harborview Medical Center (Seattle, WA, USA). From plasma obtained within 24 h of enrollment, we measured angiopoietin (Ang)-1 and Ang-2 alongside biomarkers of inflammation, including interleukin (IL)-6, IL-17 and granulocyte colony-stimulating factor. We tested for associations between standardized concentrations of biomarkers and AKI, defined by serum creatinine, from ICU admission to up to 7 days later.

**Results:**

All biomarkers of inflammation and endothelial dysfunction were associated with AKI. After adjustment for demographics, comorbidities, and IL-6 concentration, every standard deviation of Ang-1 concentration was associated with a 19 % lower risk of AKI (relative risk (RR) = 0.85, 95 % confidence interval (CI) 0.77–0.93, *p* < 0.001). Conversely, higher Ang-2 concentration was associated with higher risk of AKI (RR per standard deviation = 1.17, 95 % CI 1.13–1.22, *p* < 0.001).

**Conclusions:**

In critically ill patients, plasma concentration of the endothelial growth factors Ang-1 and Ang-2 are associated with AKI, independently of inflammation.

**Electronic supplementary material:**

The online version of this article (doi:10.1186/s13054-016-1385-3) contains supplementary material, which is available to authorized users.

## Background

Acute kidney injury (AKI) is a common complication of sepsis and the systemic inflammatory response syndrome (SIRS), and is highly associated with prolonged hospitalization, dialysis requirement, and sepsis-related death in critically ill patients [1–7]. AKI is defined as an abrupt (within 48 h) reduction in kidney function based on an elevation in serum creatinine level, a reduction in urine output, the need for renal replacement therapy (dialysis), or a combination of these factors. Unfortunately, serum creatinine is a lagging indicator of the early structural damage of AKI, rising well after the functional change in glomerular filtration rate [8–10]. This makes it difficult to prospectively identify patients at risk for severe forms of AKI and related outcomes. Novel sensitive and specific biomarkers are urgently needed to provide for cost-effective and non-invasive methods to identify those at risk for severe AKI.

For many years, neutrophil gelatinase-associated lipocalin (NGAL) was considered the most promising biomarker of AKI [11–13]. However, in the setting of critical illness its use has been hampered by a confounding lack of specificity, as the inflammatory response in sepsis causes increased synthesis of NGAL in the lungs and liver, irrespective of kidney damage [10, 14–16]. Thus, NGAL may not be a useful AKI marker in patient populations where the noise from severe systemic inflammation and multi-organ damage is so pronounced.

While the pathogenesis of AKI in critically ill patients is complex, insights into the mechanisms underlying its course may uncover novel biomarkers. Historically, severe systemic inflammation—that is, elevated circulating levels of tumor necrosis factor (TNF), interleukin (IL)-6, granulocyte colony-stimulating factor (G-CSF) and other inflammatory mediators—was thought to play the major role in sepsis-induced organ dysfunction [17–20].

However, more recently, endothelial cell activation and subsequent vascular barrier breakdown have emerged as a critical pathogenic mechanism in organ damage [21–23]. While the exact pathogenesis of AKI in this context remains unclear, recent evidence suggests an important role for microvascular endothelial injury and dysfunction in epithelial cell injury during ischemic AKI, especially during the reperfusion phase [9, 24].

Angiopoietins (Ang) are angiogenic factors essential for vascular development, maturation, and inflammation. Ang-1 and its context-dependent antagonist Ang-2 are secreted endothelial growth factors which bind to the extracellular domain of the tyrosine kinase receptor Tie2 that is predominantly expressed on endothelial cells. Ang-1 can be protective, with a role in stabilizing endothelium, while Ang-2, which promotes vascular leak, can worsen outcomes in sepsis [25–28]. With activation of the endothelium, luminal adhesion molecules are upregulated, including soluble vascular cell adhesion molecule-1 (sVCAM-1) [29].

Biomarkers of endothelial injury and dysfunction have been implicated in the development of poor outcomes in diseases with systemic inflammation [30–32]. However, the clinical significance of endothelial markers in the setting of AKI has not yet been clarified.

## Methods

### Study population

The Harborview Medical Center (Seattle, WA, USA) SIRS cohort includes patients admitted to the intensive care unit (ICU) for more than 24 h and with at least two of four SIRS criteria [33, 34]. Exclusion criteria include admission for major trauma, intracranial hemorrhage, HIV, immunosuppression, or a current diagnosis of cancer. All subjects have associated plasma specimens obtained within 24 h of admission to the ICU, and were followed until hospital discharge or death. The institutional review board of the University of Washington approved the protocols for recruitment and sample collection, which was performed with informed consent of the participants.

### Biomarker measurement

Plasma samples were thawed and concentrations of IL-6, IL-8, G-CSF, tumor necrosis factor receptor-1 (TNFR-1), Ang-1, Ang-2, and sVCAM-1 were all measured on the same day using electrochemiluminescent immunoassays (Meso Scale Discovery, Rockville, MD, USA). Samples were diluted to fit within the dynamic range of the assay, defined as the following: 0.08–2500 pg/mL for IL-6, IL-8, and TNFR-1; 0.12–5000 pg/mL for G-CSF; 3–100,000 pg/mL for Ang-1; 0.5–10,000 pg/mL for Ang-2; and 0.05–1000 pg/mL for sVCAM-1. The samples that fell below the lower or upper limits of detection were assigned those values. Missingness (~5 %) in certain biomarker concentrations was due to insufficient sample volumes in a small subset of subjects.

### Acute kidney injury

Serum creatinine was determined daily for the duration of the hospital stay as part of routine patient care. AKI status was determined by changes in serum creatinine in accordance with the Kidney Disease Improving Global Outcomes (KDIGO) criteria for AKI [35], from ICU admission to up to 7 days later. AKI was defined as any increase in serum creatinine by 0.3 mg/dL within 48 h or an increase in serum creatinine by 1.5-fold within 7 days. Severe AKI was defined as KDIGO stages 2 or 3. Because this study was not designed with AKI as a primary endpoint, hourly urine output and renal replacement therapy data were not available to provide enough granularity to the severity of AKI definition.

### Covariates

Admission data, hospital course, and complications were obtained from the electronic medical record. The APACHE (Acute Physiology, Age, Chronic Evaluation) III prognostic score and the Sequential Organ Failure Assessment (SOFA) were calculated as previously described [36, 37]. Baseline serum creatinine was defined as creatinine at admission, and chronic kidney disease (CKD) status was defined as a glomerular filtration rate below 60 mL/min/1.73 m^2^ as estimated using the creatinine-based CKD-EPI equation [38].

### Statistical analyses

Baseline descriptive statistics on demographics, medical history, and admission characteristics were examined by AKI stage. We report continuous variables as means ± standard deviations and categorical variables as numbers and percentages.

Plasma biomarker concentrations were tabulated by AKI stage and reported as median and interquartile range. *P* values were obtained using Wald tests on the grouped linear AKI stage term regressed on the log-base transformed biomarker concentration.

Pearson’s correlation coefficient (ρ) was used to estimate the magnitude of the linear correlation between log-transformed biomarker concentrations.

For associations of biomarkers with the risk of AKI, we first defined our outcome as AKI of any stage and secondarily examined associations of biomarkers with severe AKI, to increase specificity of the outcome definition. Relative risk regression was used to model the probability of AKI as a function of covariates using a generalized linear model with log link and binomial error distribution [39]. In cases in which the model failed to converge with the binomial error (~10 % of the models), we substituted Gaussian error and used robust standard error estimates. We used relative risk regression rather than logistic regression because the prevalence of AKI is not rare (occurring in >22 % of participants during hospitalization), hence the odds ratio is an overestimate of the relative risk.

Univariate and multivariable associations of biomarker concentrations with AKI were presented as relative risks (RRs) per standard deviation of the biomarker. The first adjustment model included baseline age, gender, admitting service (medical, surgical), body mass index, smoking status, prevalent diabetes, chronic renal insufficiency, and cirrhosis. Subsequent models added variables to the basic adjustment model that may possibly confound or mediate the associations of interest. The second model added the APACHE III score, and the final model added log-transformed circulating IL-6 concentrations.

We performed a secondary analysis to evaluate temporality issues and the potential of reverse causality, in which we repeated examination of the association of biomarkers with the AKI endpoint after exclusion of patients with AKI events occurring within the first 24 h of admission.

We evaluated the significance of all 32 two-way multiplicative interactions among biomarkers using the Wald test with a Bonferroni-corrected two-tailed α = 0.0016 (0.05/32). All other *p* values were two-tailed (α = 0.05) and all analyses were performed using Stata release 13.1 (College Station, TX, USA).

## Results

At baseline, the mean age was 55 years and 36 % of patients were women. Of the 948 patients admitted, 506 (53 %) experienced an AKI event, according to KDIGO criteria. As expected, patients with AKI of higher stages were more likely to have higher APACHE III scores and higher SOFA scores, and were more likely to have been admitted to the ICU from a medical source (Table 1).

**Table 1:** Baseline characteristics by AKI stage

Variable	No AKI	AKI stage 1	AKI stage 2	AKI stage 3
*N*	442	376	17	113
Age, years	55.0 (16.8)	56.8 (16.0)	47.6 (18.0)	53.0 (15.0)
BMI, kg/m^2^	28.1 (18)	32.0 (18.3)	30 (6.3)	32.4 (11.8)
Creatinine, mg/dL	0.86 (0.39)	1.14 (0.53)	0.74 (0.22)	3.74 (3.08)
SOFA score	3 (2.5)	4.7 (2.8)	3.8 (2.7)	7.5 (3.2)
APACHE III	42.2 (24.3)	55.1 (27)	53.6 (16.5)	66.5 (34.8)
Caucasian	442 (100)	376 (100)	17 (100)	113 (100)
Female gender	176 (39.8)	119 (31.7)	8 (47.1)	41 (36.3)
Source of ICU admission				
Medical	228 (51.6)	200 (53.2)	8 (47.1)	83 (73.5)
Surgical	214 (48.4)	176 (46.8)	9 (52.9)	30 (26.6)
Death within 28 days	14 (3.2)	63 (16.8)	3 (17.7)	31 (27.4)
Comorbidities				
Chronic kidney disease	12 (2.7)	34 (9.0)	0 (0)	20 (17.7)
Diabetes mellitus	86 (19.5)	111 (29.5)	7 (41.2)	30 (26.6)
Cirrhosis	36 (8.1)	35 (9.3)	3 (17.7)	10 (8.9)
Current smoking	264 (59.7)	205 (54.5)	9 (52.9)	62 (54.9)
Source of critical illness				
Pneumonia	85 (19.2)	87 (23.1)	5 (29.4)	17 (15.0)
Sepsis	269 (60.9)	269 (71.5)	14 (82.4)	80 (70.8)
Other	74 (16.7)	79 (21.0)	3 (17.7)	21 (18.6)

*AKI* acute lung injury, *APACHE* Acute Physiology, Age, Chronic Evaluation, *BMI* body mass index, *ICU* intensive care unit, *SOFA* Sequential Organ Failure Assessment

**Fig. 1:**
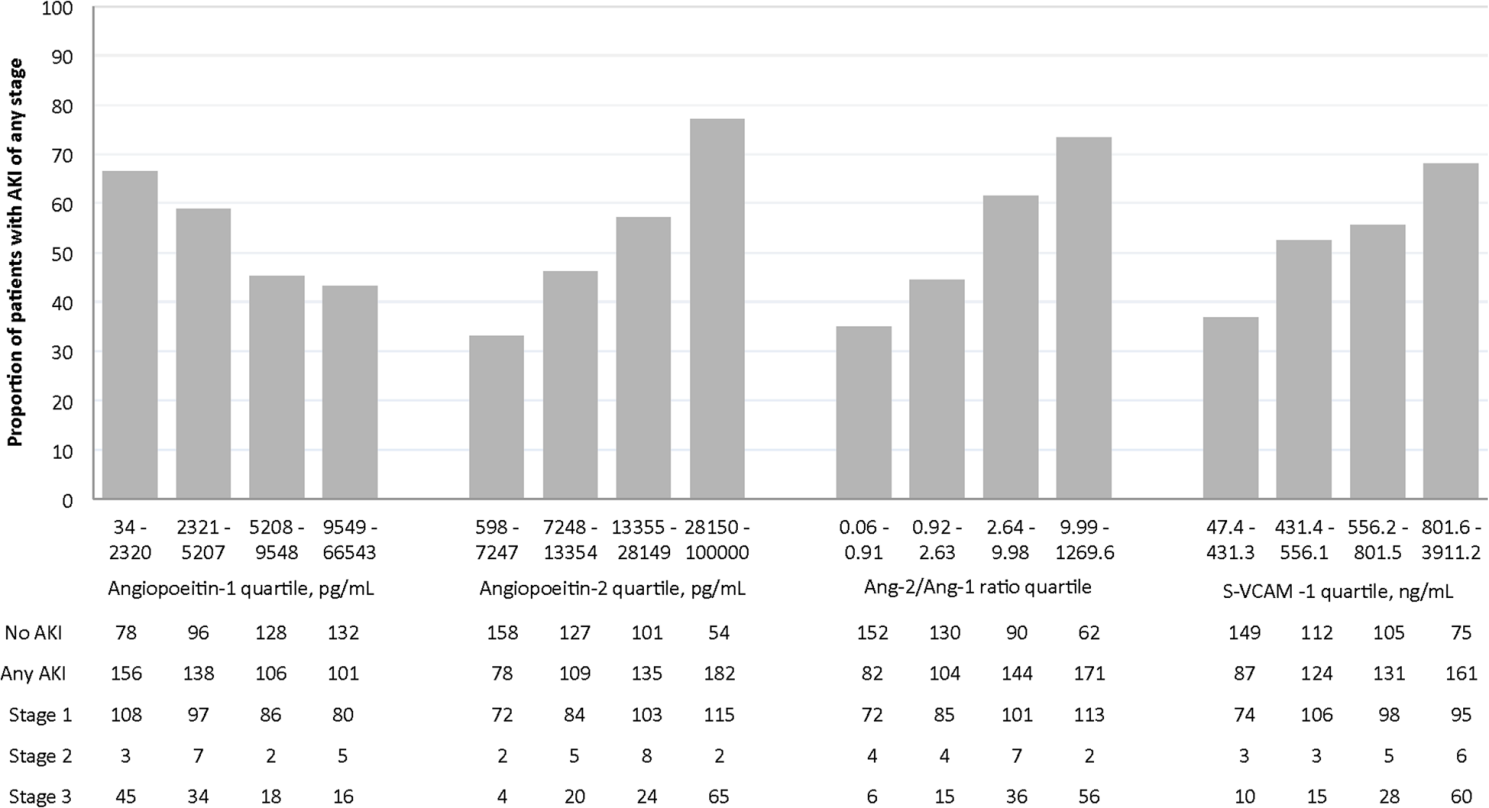
Distribution of acute kidney injury (*AKI*) cases by biomarker concentration. Bar chart displays the percentage of participants within each biomarker concentration quartile that developed AKI of any stage. Below the bar chart, the number of participants within each biomarker quartile is broken down into case status for AKI and, among those with AKI, into KDIGO AKI stage. *Ang* angiopoietin, *S-VCAM-1* soluble vascular cell adhesion molecule-1

Measured concentrations of all plasma biomarkers were associated with AKI stage (all *p* < 0.0025). Patients with higher stages of AKI had higher median concentrations of the inflammatory biomarkers IL-6, IL-8, IL-17, G-CSF, and TNFR-1, higher median concentrations of the endothelial biomarkers Ang-2, the Ang-2/Ang-1 ratio, and sVCAM-1, and lower median concentrations of Ang-1 (Table 2, Fig. 1).

**Table 2:** Plasma biomarkers according to AKI stage

Biomarker concentration	*N*	No AKI	AKI KDIGO stage			
			Stage 1	Stage 2	Stage 3	
		Median (IQR)	Median (IQR)	Median (IQR)	Median (IQR)	*p*-value
Endothelial						
Ang-1, pg/mL	935	6487.3 (3160.7–10,867.1)	4424.7 (2084.6–8424.4)	4393.3 (3458.2–13,075.6)	3209.9 (1481.1, 6574.4)	<0.0001
Ang-2, pg/mL	944	9535.4 (5740.2–17,154.9)	16283.0 (8491.6–32,757.3)	16149.8 (9764.0–22,833.7)	31227.4 (17316.6, 67176.1)	<0.0001
Ang-2/Ang-1	935	1.4 (0.65–4.3)	3.9 (1.8–13.3)	2.7 (1.1–7.6)	9.4 (3.7, 34.6)	<0.0001
sVCAM-1, ng/mL	944	497.4 (402.9–699.2)	569.9 (446.2–803.2)	708.6 (485.5–1058.6)	828.6 (601.5, 1249.9)	<0.0001
Inflammatory						
IL-6, pg/mL	891	99.8 (46.5–233.4)	151.4 (63.6–420.1)	174.5 (82.3–461.5)	171.8 (102.2, 400.9)	<0.0001
IL-8, pg/mL	891	11.0 (6.1–22.6)	14.8 (7.7–35.0)	16.4 (9.0–32.6)	22.8 (11.4, 51.0)	<0.0001
IL-17, pg/mL	853	2.5 (1.1–7.2)	3.8 (1.4–9.7)	6.8 (1.7–11.6)	4.6 (1.7, 12.1)	0.0001
G-CSF, pg/mL	891	24.1 (14.2–45.8)	30.3 (17.2–72.6)	31.7 (17.6–66.9)	31.3 (18.2, 70.6)	0.0025
TNFR-1, pg/mL	891	6281.3 (4565.8–9790.6)	9952.6 (6411.5–14,879.7)	8910.5 (5198.26–19,151.48)	31474.3 (18514.3, 47995.3)	<0.0001

*AKI* acute lung injury, *Ang* angiopoietin, *G-CSF* granulocyte colony-stimulating factor, *IL* interleukin, *IQR* interquartile range, *KDIGO* Kidney Disease Improving Global Outcomes, *TNFR-1* tumor necrosis factor receptor 1, *sVCAM-1* soluble vascular cell adhesion molecule-1

There were moderate correlations between and among the endothelial biomarkers and inflammatory biomarkers. (Additional file 1). The strongest correlations between inflammatory and endothelial biomarkers were observed for Ang-2 and TNFR-1 (ρ = 0.60) and for Ang-2 and IL-6 (ρ = 0.49).

After adjustment for demographics, lifestyle characteristics, and comorbidities, higher concentrations of all biomarkers were associated with the adjusted risk of AKI of any stage during hospitalization. Specifically, every standard deviation higher concentration of Ang-1 was associated with an estimated 19 % lower adjusted risk of AKI (95 % confidence interval (CI): 11 % lower to 33 % lower, *p* < 0.001; Table 3) and every standard deviation higher concentration of Ang-2 was associated with an estimated 22 % higher risk of AKI (95 % CI: 18 % higher to 26 % higher, *p* < 0.001; Table 3).

**Table 3:** Associations of biomarkers with acute kidney injury (any stage)

Biomarkers	Unadjusted RR (95 % CI)	Adjusted RR^a^ (95 % CI)	APACHE III-adjusted RR^b^ (95 % CI)	IL6-adjusted RR^c^ (95 % CI)
Endothelial				
Ang-1	0.81 (0.74–0.88)***	0.81 (0.73–0.89)***	0.87 (0.80–0.95)**	0.85 (0.77–0.93)***
Ang-2	1.22 (1.19–1.25)***	1.22 (1.18–1.26)***	1.16 (1.11–1.20)***	1.17 (1.13–1.22)***
Ang-2/Ang-1	1.06 (1.03–1.08)***	1.06 (1.03–1.08)***	1.04 (1.02–1.06)***	1.05 (1.03–1.07)***
sVCAM-1	1.09 (1.07–1.12)***	1.17 (1.12–1.21)***	1.13 (1.07–1.18)***	1.14 (1.10–1.19)***
Inflammatory				
IL-6	1.10 (1.07–1.14)***	1.10 (1.06–1.14)***	1.04 (1.00–1.08)	–
IL-8	1.04 (1.01–1.07)*	1.04 (1.02–1.07)**	1.01 (0.98–1.03)	1.01 (0.98–1.04)
IL-17	1.07 (1.05–1.10)***	1.06 (1.03–1.09)***	1.05 (1.02–1.08)**	1.04 (1.00–1.07)*
G-CSF	1.06 (1.03–1.10)**	1.06 (1.03–1.10)**	1.01 (0.97–1.06)	0.99 (0.95–1.04)
TNFR-1	1.21 (1.18–1.23)***	1.20 (1.17–1.22)***	1.16 (1.14–1.19)***	1.17 (1.15–1.20)***

Relative risks (RR) presented per standard deviation of each biomarker

^a^Relative risk regression adjusted for age, gender, sepsis, admitting service (ex: medical = 1, surgical = 0), body mass index, smoking status, diabetes mellitus, chronic renal insufficiency, and cirrhosis

^b^Adjusted for APACHE III and covariates in ^a^

^c^Adjusted for Log_2_ (IL-6) concentration and covariates in ^a^

*p < 0.05; **p < 0.01; ***p < 0.001

*Ang* angiopoietin, *APACHE* Acute Physiology, Age, Chronic Evaluation, *CI* confidence interval, *G-CSF* granulocyte colony-stimulating factor, *IL* interleukin, *TNFR-1* tumor necrosis factor receptor 1, *sVCAM-1* soluble vascular cell adhesion molecule-1

Similarly, higher concentrations of all endothelial and inflammatory biomarkers were associated with the risk of stage 2–3 AKI during hospitalization. Specifically, every standard deviation higher concentration of Ang-1 was associated with an estimated 50 % lower adjusted risk of stage 2–3 AKI (95 % CI: 33 % lower to 67 % lower, *p* = 0.002; Additional file 2) and every standard deviation higher concentration of Ang-2 was associated with an estimated 59 % higher risk of stage 2–3 AKI (95 % CI: 48 % higher to 72 % higher, *p* < 0.001; Additional file 2). Associations of endothelial biomarkers with risk of stage 2–3 AKI were robust to adjustment for the APACHE III score and for circulating IL-6.

In sensitivity analyses that excluded 173 participants with AKI events within the first 24 h of admission, associations were similar and more robustly similar for the endothelial biomarkers (Additional file 3). Significant two-way interactions (*p* < 0.001) among the biomarkers in association with AKI events were observed for the Ang-2/Ang-1 ratio and IL-17, VCAM, Ang-2, and G-CSF, and for sVCAM with TNFR-1 and Ang-2, and for Ang-2 with TNFR-1. In a multivariable model with adjustment for all inflammatory markers and Model 1 covariates, IL-6 and TNFR-1 each remained associated with the risk of AKI. In a multivariable model with adjustment for all endothelial markers and Model 1 covariates, Ang-1 and Ang-2 each remained associated with the risk of AKI.

## Discussion

This study is the first to report associations of endothelial biomarkers with AKI in a large general medical/surgical ICU population. The results observed are consistent with previous studies, which have shown inflammatory markers to be associated with AKI events in various critically ill populations [40]. Several relatively smaller studies have recently explored associations of endothelial biomarkers and AKI. Recent case-control studies of cardiac surgery patients have found that plasma levels of Ang-2 increase post-surgically by a greater extent in patients who develop AKI than in controls [41, 42]. A cross-sectional study of critically ill patients at inception of renal replacement therapy in the ICU found that circulating Ang-2 was correlated with AKI stage and with 28-day mortality risk [43].

Accumulating evidence suggests that systemic inflammation and endothelial activation underlie the development of AKI [41, 43–46]. TNF is an inflammatory marker released by activated macrophages, monocytes, and neutrophils, and has been shown to have a major role in both sepsis and septic AKI [20, 47–49]. Renal endothelial cells are activated by TNF, further perpetuating the pro-inflammatory state and potentially sensitizing kidney tissue to subsequent damage [47, 48]. Recent efforts in animal models have demonstrated that Ang-1 may enhance the protective capacity of early endothelial outgrowth cells in murine AKI [50, 51]. Acute endothelial cell changes may lead to altered vascular reactivity, permeability, adherence of leukocytes, coagulation, and microvascular vasomotor autoregulation, perpetuating AKI.

Among a large cohort of critically ill patients admitted to the ICU, we found that circulating concentrations of inflammatory and endothelial biomarkers were significantly associated with a higher risk of AKI.

These biomarkers may highlight novel pathways of kidney injury in the setting of critical illness as well as the potential use of baseline biomarker profiles to identify individuals at risk of developing AKI. Our observations support the hypothesis that, before overt renal cellular injury has occurred, there may be alterations in microcirculation and tissue oxygenation that predispose individuals to renal damage [52]. This concept is especially relevant to the SIRS state, where inflammation and endothelial cell activation are prominent [16]. The microvasculature and endothelial cells in particular regulate blood flow to local tissue beds and modulate coagulation, inflammation, and vascular permeability. AKI has profound effects on the renal endothelium, resulting in microvascular dysfunction leading to ongoing ischemic conditions and further injury following the initial insult [53, 54].

While we highlight, for the first time, the independent association of endothelial biomarkers with AKI in critically ill patients, our study has some limitations. The most important limitation of this observational study is the potential for confounding, because characteristics such as illness severity are likely linked both with endothelial dysregulation and risk of AKI. Substantial efforts were made to adjust for potential confounding, but a causal association between the dysfunction of endothelial cells and AKI development cannot be established from our results. Second, this study was not designed with AKI as a primary endpoint and, as such, did not collect sufficiently detailed urine output data; AKI was only assessed retrospectively by changes in creatinine [34]. Data were not available on renal replacement therapy, which would have provided additional granularity to the severity of AKI definition. Moreover, sepsis decreases production of creatinine, limiting the use of changes in creatinine levels as a marker of AKI [55]. A small proportion of patients had insufficient sample volumes to measure all biomarkers simultaneously. These measurement issues may have resulted in non-differential misclassification, leading to an attenuation of associations toward the null. Third, we cannot exclude the possibility of reverse causality, wherein the damaged kidney itself may release endothelial markers before creatinine rises, and thus contributes to higher circulating levels of Ang-2 and lower circulating levels of Ang-1 [56]. Lastly, the subjects included in this study are all recruited from a single hospital and were all Caucasian, and this may limit the ability to more broadly interpret our results. We did, however, include subjects transferred from outside hospitals in the analyses and adjustment for this factor did not modify the associations with poor outcome.

## Conclusions

In conclusion, endothelial and inflammatory markers were significantly associated with AKI among patients in critical care. Further studies are needed to investigate the prognostic value of these biomarkers and to potentially identify interventions that modify these biomarkers.

## Abbreviations

AKI, acute kidney injury; Ang, angiopoietin; APACHE, Acute Physiology, Age, Chronic Evaluation; CI, confidence interval; CKD, chronic kidney disease; G-CSF, granulocyte colony-stimulating factor; ICU, intensive care unit; IL, interleukin; KDIGO, Kidney Disease Improving Global Outcomes;NGAL, neutrophil gelatinase-associated lipocalin; RR, relative risk; SIRS, systemic inflammatory response syndrome; SOFA, Sequential Organ Failure Assessment; sTNFR-1, tumor necrosis factor receptor 1; sVCAM-1, soluble vascular cell adhesion molecule-1; TNF, tumor necrosis factor

## Additional files

Additional file 1: Pairwise correlation matrix of endothelial and inflammatory biomarkers. Pearson’s correlation coefficient (ρ) was used to estimate the magnitude of the linear correlation between log-transformed biomarker concentrations. (DOCX 15 kb) Additional file 2: Associations of biomarkers with AKI (Stage 2–3 versus no AKI). For associations of biomarkers with the risk of AKI, we examined associations of biomarkers with severe AKI, to increase specificity of the outcome definition. (DOCX 15 kb) Additional file 3: Associations of biomarkers with AKI (any stage) after exclusion of 173 patients with AKI within the first 24 h of admission. We performed a secondary analysis to evaluate temporality issues and the potential of reverse causality, in which we repeated examination of the association of biomarkers with the AKI endpoint after exclusion of patients with AKI events occurring within the first 24 h of admission. (DOCX 15 kb)
