# Supplementary material for: Association of markers of endothelial dysregulation Ang1 and Ang2 with acute kidney injury in critically ill patients
Source: Crit Care. 2016 Jul 3;20:207. doi: 10.1186/s13054-016-1385-3 (PMC4930837; doi:10.1186/s13054-016-1385-3)
Supplement: Additional file 1: — Pairwise correlation matrix of endothelial and inflammatory biomarkers. Pearson’s correlation coefficient (ρ) was used to estimate the magnitude of the linear correlation between log-transformed biomarker concentrations. (DOCX 15 kb) [file 13054_2016_1385_MOESM1_ESM.docx]

Additional file 1. Pairwise correlation matrix

|  | *Endothelial markers* | | | | *Inflammatory markers* | | | |
| --- | --- | --- | --- | --- | --- | --- | --- | --- |
|  | Ang-1 | Ang-2 | Ang-2/1 | sVCAM-1 | IL-6 | IL-8 | IL-17 | G-CSF |
| *Endothelial* |  |  |  |  |  |  |  |  |
| Ang-1 | 1 |  |  |  |  |  |  |  |
| Ang-2 | -0.3289 | 1 |  |  |  |  |  |  |
| Ang-2/Ang-1 | -0.8346 | 0.7948 | 1 |  |  |  |  |  |
| sVCAM-1 | -0.3776 | 0.4776 | 0.5203 | 1 |  |  |  |  |
| *Inflammatory* |  |  |  |  |  |  |  |  |
| IL-6 | -0.1705 | 0.4878 | 0.3984 | 0.2223 | 1 |  |  |  |
| IL-8 | -0.0183 | 0.4002 | 0.2476 | 0.3216 | 0.4744 | 1 |  |  |
| IL-17 | -0.2069 | 0.3662 | 0.3476 | 0.2679 | 0.3317 | 0.197 | 1 |  |
| G-CSF | -0.1455 | 0.3338 | 0.2891 | 0.0512 | 0.6611 | 0.4579 | 0.2787 | 1 |
| sTNFR-1 | -0.2192 | 0.6043 | 0.496 | 0.524 | 0.5042 | 0.4805 | 0.2863 | 0.3054 |
