# Supplementary material for: Association of markers of endothelial dysregulation Ang1 and Ang2 with acute kidney injury in critically ill patients
Source: Crit Care. 2016 Jul 3;20:207. doi: 10.1186/s13054-016-1385-3 (PMC4930837; doi:10.1186/s13054-016-1385-3)
Supplement: Additional file 2: — Associations of biomarkers with AKI (Stage 2–3 versus no AKI). For associations of biomarkers with the risk of AKI, we examined associations of biomarkers with severe AKI, to increase specificity of the outcome definition. (DOCX 15 kb) [file 13054_2016_1385_MOESM2_ESM.docx]

Additional file 2. Associations of Biomarkers with AKI (Stage 2-3 vs. No AKI)

| Biomarker (SD) | Unadjusted RR (95%CI) | Adjusted RR**^*^** (95%CI) | Apache III-Adjusted RR^†^  (95%CI) | IL6 Adjusted RR^‡^ (95%CI) |
| --- | --- | --- | --- | --- |
| *Endothelial* |  |  |  |  |
| Ang-1 | 0.56 (0.44, 0.72)*** | 0.50 (0.33, 0.77)** | 0.71 (0.50, 1.02) | 0.59 (0.40, 0.87)** |
| Ang-2 | 1.62 (1.52, 1.73)*** | 1.59 (1.48, 1.72)*** | 1.42 (1.26, 1.60)*** | 1.52 (1.38, 1.66)*** |
| Ang-2/Ang-1 | 1.09 (1.07, 1.12)*** | 1.10 (1.07, 1.13)*** | 1.07 (1.05, 1.09)*** | 1.09 (1.06, 1.12)*** |
| sVCAM-1 | 1.26 (1.21, 1.30)*** | 1.4 (1.31, 1.51)*** | 1.29 (1.14, 1.46)*** | 1.39 (1.30, 1.49)*** |
| *Inflammatory* |  |  |  |  |
| IL-6 | 1.18 (1.05, 1.32)** | 1.13 (0.99, 1.29) | 0.93 (0.83, 1.05) | - |
| IL-8 | 1.05 (0.94, 1.18) | 1.05 (0.96, 1.16) | 0.95 (0.84, 1.08) | 0.96 (0.83, 1.10) |
| IL-17 | 1.22 (1.11, 1.35)*** | 1.02 (0.83, 1.25) | 1.06 (0.92, 1.23) | 1.04 (0.91, 1.18) |
| G-CSF | 1.06 (0.91, 1.23) | 1.05 (0.91, 1.22) | 0.87 (0.73, 1.04) | 0.87 (0.66, 1.13) |
| sTNFR-1 | 1.44 (1.40, 1.49)*** | 1.44 (1.39, 1.49)*** | 1.40 (1.34, 1.45)*** | 1.42 (1.36, 1.47)*** |

Relative risks presented per standard deviation of each biomarker.

**^*^** Relative risk regression adjusted for age, gender, sepsis, admitting service (ex: medical = 1, surgical = 0), body mass index, smoking status, diabetes mellitus, chronic renal insufficiency, and cirrhosis

^†^ Adjusted for APACHE III and covariates in ^*^.

^‡^ Adjusted for Log_2_(IL-6) concentration and covariates in ^*^.

***p<0.001; **p<0.01; *p<0.05
