# Supplementary material for: Association of markers of endothelial dysregulation Ang1 and Ang2 with acute kidney injury in critically ill patients
Source: Crit Care. 2016 Jul 3;20:207. doi: 10.1186/s13054-016-1385-3 (PMC4930837; doi:10.1186/s13054-016-1385-3)
Supplement: Additional file 3: — Associations of biomarkers with AKI (any stage) after exclusion of 173 patients with AKI within the first 24 h of admission. We performed a secondary analysis to evaluate temporality issues and the potential of reverse causality, in which we repeated examination of the association of biomarkers with the AKI endpoint after exclusion of patients with AKI events occurring within the first 24 h of admission. (DOCX 15 kb) [file 13054_2016_1385_MOESM3_ESM.docx]

Additional file 3. Associations of Biomarkers with AKI (any stage) after exclusion of 173 patients with AKI within the first 24 hours of admission

| Biomarkers (SD) | Unadjusted RR (95%CI) | Adjusted RR (95%CI) | Apache III Adjusted RR (95%CI) | IL6 Adjusted RR (95%CI) |
| --- | --- | --- | --- | --- |
| *Endothelial* |  |  |  |  |
| Ang-1 | 0.83 (0.74, 0.93)*** | 0.82 (0.71, 0.95)** | 0.87 (0.76, 1.01) | 0.83 (0.72, 0.96)* |
| Ang-2 | 1.21 (1.17, 1.26)*** | 1.20 (1.14, 1.25)*** | 1.15 (1.09, 1.21)*** | 1.15 (1.09, 1.22)*** |
| Ang-2/Ang-1 | 1.09 (1.06, 1.12)*** | 1.10 (1.06, 1.14)*** | 1.08 (1.04, 1.11)*** | 1.10 (1.06, 1.14)** |
| sVCAM-1 | 1.10 (1.06, 1.15)*** | 1.16 (1.09, 1.24)*** | 1.13 (1.06, 1.21)*** | 1.14 (1.08, 1.20)*** |
| *Inflammatory* |  |  |  |  |
| IL-6 | 1.10 (1.05, 1.15)*** | 1.10 (1.04, 1.16)*** | 1.04 (0.97, 1.12) | - |
| IL-8 | 1.01 (0.96, 1.05) | 1.01 (0.97, 1.06) | 0.98 (0.94, 1.04) | 0.98 (0.94, 1.03) |
| IL-17 | 1.06 (1.02, 1.10)*** | 1.06 (1.03, 1.09)** | 1.05 (1.00, 1.10) | 1.04 (1.00, 1.07) |
| G-CSF | 1.05 (0.99, 1.10) | 1.05 (0.99, 1.11) | 1.00 (0.93, 1.07) | 0.99 (0.93, 1.11) |
| sTNFR-1 | 1.15 (1.12, 1.19)*** | 1.14 (1.11, 1.18)*** | 1.13 (1.09, 1.16)*** | 1.11 (1.07, 1.15)*** |

Relative risks presented per standard deviation of each biomarker.

**^*^** Relative risk regression adjusted for age, gender, sepsis, admitting service (ex: medical = 1, surgical = 0), body mass index, smoking status, diabetes mellitus, chronic renal insufficiency, and cirrhosis

^†^ Adjusted for APACHE III and covariates in ^*^.

^‡^ Adjusted for Log_2_(IL-6) concentration and covariates in ^*^.

***p<0.001; **p<0.01; *p<0.05
